# The influences of walking, running and stair activity on knee articular cartilage: Quantitative MRI using T1 rho and T2 mapping

**DOI:** 10.1371/journal.pone.0187008

**Published:** 2017-11-14

**Authors:** Meng Chen, Lin Qiu, Si Shen, Fei Wang, Jing Zhang, Cici Zhang, Sirun Liu

**Affiliations:** Medical Imaging Center, The First Affiliated Hospital of Jinan University, Tianhe District, Guangzhou City, Guangdong Province, China; University Hospital Modena and Reggio Emilia, ITALY

## Abstract

**Objective:**

To explore the different influences of walking, running and stair activity on knee articular cartilage with T1 rho and T2 mapping sequences.

**Materials and methods:**

MRI (3.0-T) scans of the right knee were performed in twenty-three young healthy adults immediately after 30 minutes of rest, walking, running and stair activity respectively. Articular cartilage was quantitatively assessed based on T1 rho and T2 relaxation times. Analysis of variance for random block design data, bonferroni test and paired samples t tests were performed to estimate the different influences of physiological activities on articular cartilage.

**Results:**

T1 rho and T2 values had reductions after physiological activities in all regions of articular cartilage. T1 rho and T2 values were decreased more after running than walking. T1 rho and T2 values were decreased more after stair activity than running, except for femoral cartilage. The superficial layer of patella cartilage had higher reduction rates than the deep layer. The T1 rho and T2 values of articular cartilage were reduced in the following order: patellofemoral cartilage> medial tibiofemoral cartilage> lateral tibiofemoral cartilage. Patellofemoral cartilage experienced reductions in the following order: lateral part> middle part> medial part. Tibiofemoral cartilage had reductions in the following order: posterior part> middle part> anterior part.

**Conclusions:**

T1 rho and T2 mapping sequences can quantitatively reflect the different influences of physiological activities on knee articular cartilage. Fluid shifts, collagen fiber deformation, spatial heterogeneity, inherent differences in material properties and tissue stiffness have close relationship with cartilage loading characteristics.

## Introduction

Biomechanically, articular cartilage is important for shock absorption and load transmission. Articular cartilage can transfer physiological loads to the subchondral bone and reduce friction within the joint.

Many researchers have explored the relationship between sports and knee cartilage injury, degeneration and osteoarthritis. Relevant studies [1–7] have shown that sports influence the biochemical composition and micromorphology of cartilage, resulting in cartilage deformation. Some studies seem to indicate that moderate levels of running do not increase the risk of osteoarthritis (OA) in healthy people [8,9]. Some researchers have reported that high-impact sports involving repetitive joint loading (such as running) are a biomechanical risk factor for developing knee osteoarthritis [9,10]. Long-term chronic joint loading and excessive exercise lead to intra-articular injury and deformation, thus causing osteoarthritis or accelerating the process of osteoarthritis [11–13]. Subburaj et al [5] explored the acute effect of running using T1 rho and T2 mapping images, and the T1 rho and T2 values were reduced after running. Souza [6] observed decreases in the T1 rho and T2 values of cartilage under simulated static loading in vivo. However Luke et al [7] showed that cartilage T1 rho and T2 values are higher in long–distance runners after running a marathon.

In physiological activities, the possibility of degeneration and injury in the knee articular cartilage exhibits anatomical regional variation and spatial heterogeneity due to the differences in load distribution. Additionally, inherent differences in local material properties are also key factor. Hence, the ability to spatially localize the effect of exercise on cartilage and to explore anatomic differences in material properties of articular cartilage are necessary to understand the mechanism behind cartilage degeneration and injury. It is unclear what influences these different loading patterns have on different anatomical regions of knee cartilage. Although substantial data on knee kinematics is available for walking and running, much less data are available on knee biomechanics during stair activity.

In recent years, MR quantitative techniques have provided a microscopic view of the biochemistry and function of cartilage. T1 rho-weighted imaging and T2 mapping are new magnetic resonance (MR) quantitative technologies that are sensitive to changes in proteoglycan (PG), collagen and water content.

The purposes of this study were to investigate the different influences of walking, running and stair activity on knee articular cartilage with T1 rho and T2 mapping sequences. The research would be valuable in sports medicine, biomechanics and sport-related osteoarthritis, and lay a foundation for sports training after cartilage injury.

## Materials and methods

The research was approved by the Ethics Committee for Human Research at The First Affiliated Hospital of Jinan University (The approval number: [2015] 060). All participants signed an informed consent form.

Thirty young healthy adults between 20 and 30 years of age with no knee trauma, no pain or discomfort, and no history of knee surgery were recruited. The maximum age of 30 years was selected to avoid cases of undiagnosed or early osteoarthritis. All participants had no contraindications for undergoing MR scans (implanted pacemaker, the presence of metal, etc.).

Among the 30 volunteers, 4 volunteers had obvious artifacts, rendering the data inaccurate. 3 volunteers suffered injury among the experiment (1 volunteer suffered anterior cruciate ligament injury in football match. 1 volunteer had patella edema by falling from the bicycle and 1 volunteer experienced knee pain without abnormal signal). Finally, 23 volunteers were included (11 males and 12 females) with an average age of 25 years of age (range: 23–30 years) and an average body mass index (BMI) of 20.29 Kg/m^2^ (range: 16.02–24.77 Kg/m^2^).

MRI (3.0 T) scans of the right knee were performed in the participants immediately after 30 minutes rest and walking respectively. The 30 minutes rest was used to reduce the influence of previous physical activities on MR relaxation times in cartilage. After one week, 3.0-T MRI scans were performed after 30 minutes running; after two weeks, the same scans were performed after 30 minutes stair activity. According to the guidelines of the new American College of Sports Medicine, adults aged from 18 to 65 years should perform at least 30 minutes of moderate-intensity aerobic physical activity 5 days each week to protect against some chronic diseases [5]. During the rest period and all MR scans, the volunteers were placed in a supine position, and their lower extremity was placed in neutral rotation with the knee in full extension.

During walking, running and stair activity, the distance (Km), speed (Km/h) and number of calories (Kcal) burned were recorded. Walking and running were performed on a treadmill. The average speed of walking was 6.1 Km/h (range: 4.9–7.4 Km/h), and the average number of calories burned was 186.3 Kcal (range: 146.7–223.3 Kcal). The average speed of running was 10.4 Km/h (range: 8.4–11.6 Km/h), and the average number of calories burned was 313.8 Kcal (range: 252.4–347.8 Kcal). There were 28 steps per floor of stair activity, and the height and the width of the steps were 15 cm and 28 cm, respectively. The average stride frequency during the stair activity was 111.8 strides/min (range: 100.0–131.0 strides/min), and the average number of calories burned was 209.2 Kcal (range: 182.4–259.0 Kcal).

### Magnetic resonance imaging (MRI) protocol

All imaging was performed using a 3.0-T GE discovery MR750 scanner and an eight-channel transmit-receive phased array knee coil. The imaging protocol included sagittal proton density-weighted, fat-saturated, fast spin-echo (PD-FS-FSE) images (repetition time [TR]/echo time [TE] = 3376/30 ms, field of view [FOV] = 14 cm, matrix = 384×256, slice thickness = 2.5 mm, gap = 0.5 mm, echo train length [ETL] = 8, number of excitations [NEX] = 2, Bandwidth = 31.25 kHz), sagittal 3-dimensional fat-saturated, high-resolution, spoiled gradient-echo (3D-FS-SPGR) images (TR/TE = 14.8 ms/Min Full, FOV = 14 cm, matrix = 384×256, slice thickness = 3.0 mm, Flip Angle = 15°, NEX = 1, Bandwidth = 31.25 kHz), sagittal and axial 3-dimensional T1 rho-weighted images (TR/TE = 6.1 ms/Min Full, FOV = 14 cm, matrix = 288×192, slice thickness = 3.0 mm, NEX = 1, Bandwidth = 41.67 kHz, Spin Lock Frequency = 500.00 Hz, TSL (1–4) = 0, 10, 40, 80 ms, T1 Recovery Time = 1200 ms), and sagittal and axial T2 mapping images (TR = 2400 ms, FOV = 14 cm, matrix = 256×160, slice thickness = 2.5 mm, gap = 0.5 mm, ETL = 8, NEX = 1, Bandwidth = 83.33 kHz, TE per Scan = 8).

### Cartilage segmentation, data measurement and data integration

**Fig 1:**
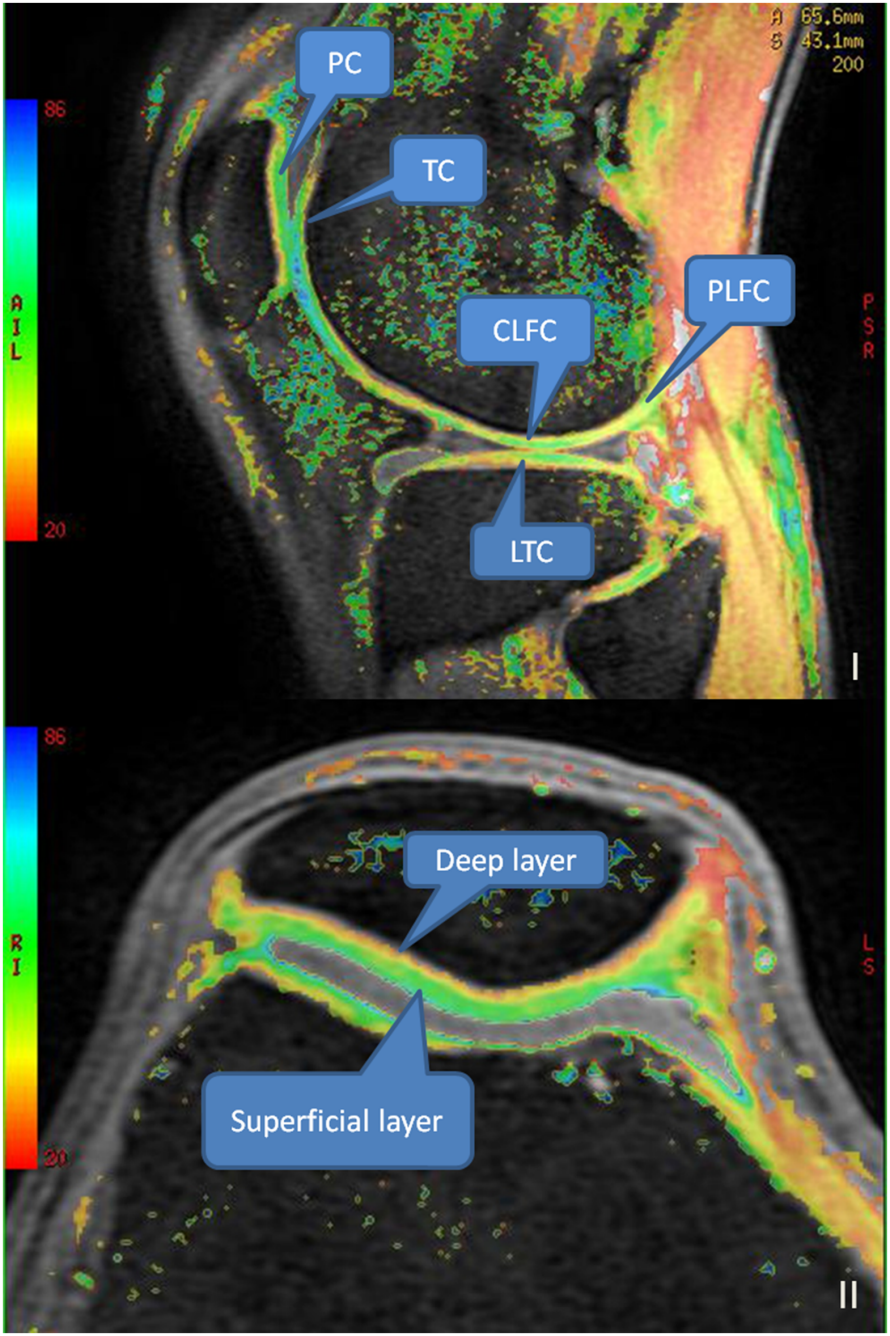
I The segmentation of knee cartilage; II The superficial and deep layers of patella cartilage. Note: PC = patella cartilage, TC = trochlea cartilage, LFC = lateral femoral cartilage, LTC = lateral tibial cartilage.

Cartilage was divided into 6 regions: medial and lateral femoral cartilage (MFC and LFC, respectively), medial and lateral tibial cartilage (MTC and LTC, respectively), and patella and trochlea cartilage (PC and TC, respectively). Patella cartilage and trochlea cartilage were further divided into 3 parts: medial, middle and lateral. Patella cartilage was further divided into superficial and deep layers. Femoral cartilage was partitioned into central weight-bearing regions (CMFC/CLFC) and posterior nonweight-bearing regions (PMFC/PLFC) with the posterior boundary of the meniscus as the dividing mark. The central weight-bearing regions of the femoral cartilage and the tibial cartilage were divided into 3 parts: anterior, middle and posterior. The segmentation was shown in Fig 1.

Two musculoskeletal radiologists had a systematic, blinded and independent evaluation towards all MR images. PD-FS-FSE images and 3D-FS-SPGR images were used to evaluate internal degeneration, meniscal tears and cartilage defects. The collected T1 rho and T2 mapping images were processed on an Advantage Workstation version 4.5 image post-processing workstation, and T1 rho and T2 mapping pseudo-color maps were generated by merging the PD-FS-FSE images with T1 rho-weighted images or T2 mapping images using Functool post-processing software (Fig 1). The thickest layers of the cartilage were selected to measure the T1 rho and T2 values with S_ROI_ 2–4 mm^2^. the measurements avoided bone cortex, meniscus and joint fluid to reduce their impact on the measured relaxation times. The selection and measurement of ROI were greatly influenced by subjective factors, so the same radiologist made two measurements at different times (more than 1 month), and a consistency t test was performed.

To analyze the different effects of the exercises and the biomechanical mechanisms of patellofemoral and femorotibial cartilage, knee articular cartilage was merged into three larger groups: patella cartilage and trochlea cartilage were grouped as patellofemoral cartilage; medial femoral cartilage and medial tibial cartilage were grouped as medial tibiofemoral cartilage; and lateral femoral cartilage and lateral tibial cartilage were grouped as lateral tibiofemoral cartilage. Furthermore, patellofemoral cartilage was divided into medial, middle and lateral parts, and tibiofemoral cartilage was divided into anterior, middle and posterior parts.

### Statistical analysis

Statistical analysis was performed using SPSS version 19.0 at a significance level of P<0.05. The statistical data were presented as the means ± standard deviations. Analysis of variance (ANOVA) for random block design data was performed to analyse whether the influence of walking, running and stair activity on different parts of the knee articular cartilage had statistical difference and further, a Bonferroni test was used to estimate the different changes of three different exercises on knee articular cartilage. A paired t test was conducted to evaluate the different changes on T1 rho and T2 relaxation times of superficial and deep layers of patella cartilage.

## Results

**Fig 2:**
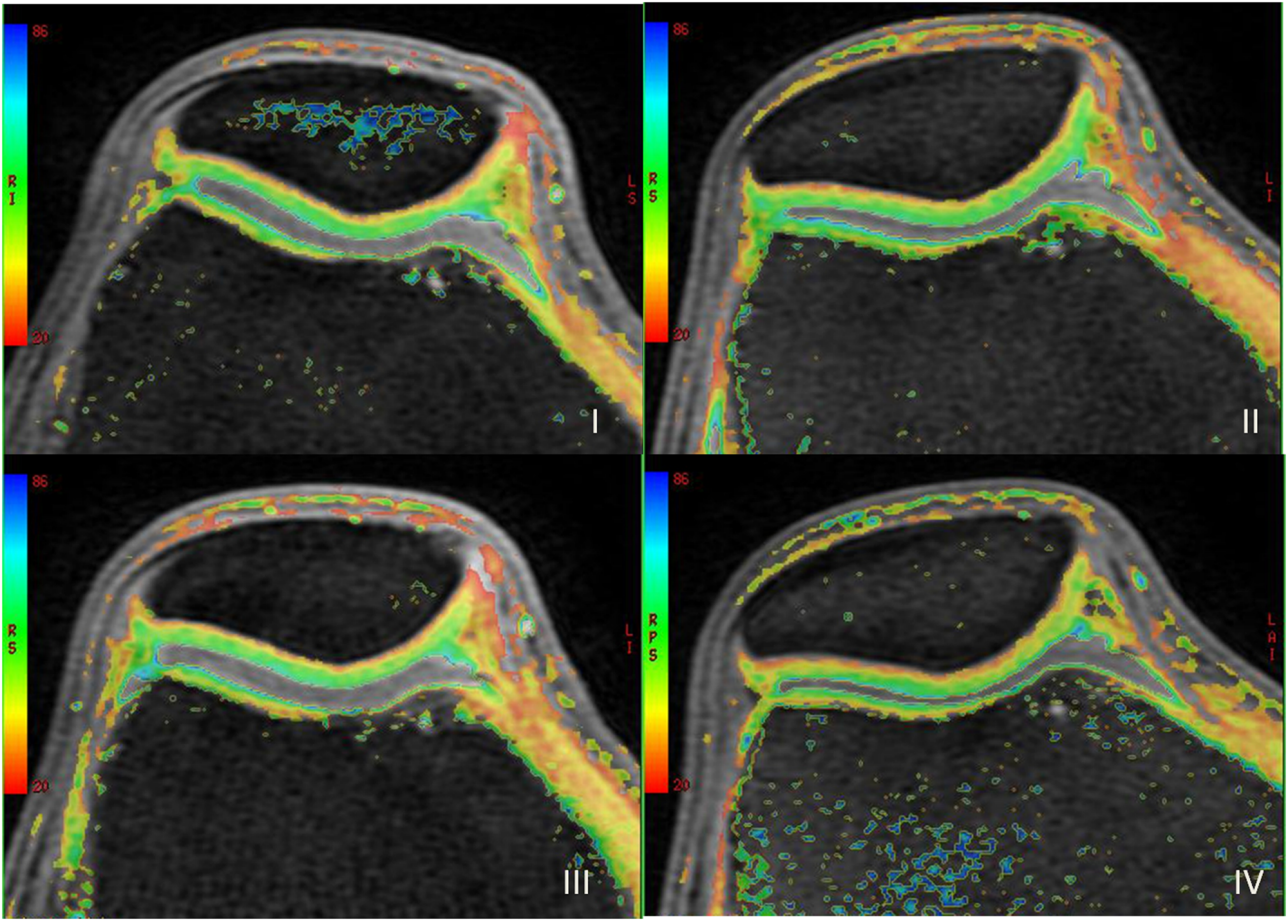
The T1 rho pseudo color maps after 30 minutes rest(I), walking(II), running(III) and stair activity(IV) presented the color of cartilage in a range from green to yellow for the superficial layer and from yellow to red in the deep layer.

No obvious differences in PDWI, 3D-FS-SPGR, T1 rho and T2 mapping original images were found in cartilage between the exercises and rest samples. T1 rho and T2 mapping pseudo-color maps presented the color of cartilage in a range from green to yellow for the superficial layer and from yellow to red in the deep layer (Fig 2). T1 rho and T2 values after physiological activities were lower than rest values for all regions of knee cartilage. T1 rho and T2 values were decreased more after running than walking. T1 rho and T2 values were decreased more after stair activity than running, except for the middle and posterior parts of the medial femoral cartilage and the posterior part of the lateral femoral cartilage.

### Patellofemoral cartilage

According to an analysis of variance for random block design data (Table 1), the T1 rho values of patella and trochlea cartilage after walking, running and stair activity significantly differed (P<0.05); the T2 values also significantly differed, except for the deep layer of medial patella cartilage.

**Table 1:** ANOVA for random block design data and bonferroni test of patellofemoral cartilage among rest, walking, running and stair activity.

Cartilage	T1 rho								T2 mapping							
	F value	P value	1 vs 2	1 vs 3	1 vs 4	2 vs 3	2 vs 4	3 vs 4	F value	P value	1 vs 2	1 vs 3	1 vs 4	2 vs 3	2 vs 4	3 vs 4
Superficial layer of PC																
Medial part	46.49	<0.001	0.037	<0.001	<0.001	<0.001	<0.001	0.244	36.62	<0.001	0.011	<0.001	<0.001	0.001	<0.001	0.193
Middle part	86.43	<0.001	<0.001	<0.001	<0.001	<0.001	<0.001	0.161	30.39	<0.001	0.001	<0.001	<0.001	0.013	<0.001	0.468
Lateral part	327.50	<0.001	<0.001	<0.001	<0.001	<0.001	<0.001	<0.001	94.92	<0.001	<0.001	<0.001	<0.001	<0.001	<0.001	<0.001
Deep layer of PC																
Medial part	8.81	<0.001	1.000	0.063	<0.001	0.779	0.002	0.183	2.67	0.055	0.744	0.050	0.277	1.000	1.000	1.000
Middle part	20.81	<0.001	0.401	<0.001	<0.001	0.002	<0.001	1.000	3.81	0.014	0.291	0.106	0.011	1.000	1.000	1.000
Lateral part	34.35	<0.001	0.003	<0.001	<0.001	0.006	<0.001	0.121	7.09	<0.001	0.021	0.025	<0.001	1.000	0.895	0.807
TC																
Medial part	43.03	<0.001	0.035	<0.001	<0.001	<0.001	<0.001	1.000	17.24	<0.001	0.034	<0.001	<0.001	0.150	0.001	0.687
Middle part	41.34	<0.001	0.001	<0.001	<0.001	0.002	<0.001	0.103	30.42	<0.001	<0.001	<0.001	<0.001	0.023	<0.001	0.812
Lateral part	146.10	<0.001	<0.001	<0.001	<0.001	<0.001	<0.001	<0.001	103.44	<0.001	<0.001	<0.001	<0.001	<0.001	<0.001	0.004

Note: PC = patella cartilage, TC = trochlea cartilage;

1: rest, 2: walking, 3: running, 4: stair activity.

According to the Bonferroni test for comparison of multiple samples(Table 1), the T1 rho values for patella and trochlea cartilage differed significantly (P<0.05) after walking, except for the deep layer of medial and middle patella cartilage; the T1 rho values for patella and trochlea cartilage differed significantly (P<0.05) after running, except for the deep layer of medial patella cartilage; and the T1 rho values for patellofemoral cartilage differed significantly (P<0.05) after stair activity. The T2 value differed significantly (P<0.05) after walking, except for the deep layer of medial and middle patella cartilage; the T2 value differed significantly (P<0.05) after running, except for the deep layer of medial and middle patella cartilage; and the T2 value differed significantly (P<0.05) after stair activity, except for the deep layer of medial patella cartilage.

The T1 rho and T2 relaxation times of the superficial layer were longer than those of the deep layer of patella cartilage, and the differences were significant (T1 rho mean value:46.772 ms vs 35.547 ms, P<0.001; T2 mean value:42.934 ms vs 31.473 ms, P<0.001). Compared with rest values, the T1 rho and T2 values of the superficial layers after physiological activities decreased more than the deep layers. The reduction rates of superficial layer after walking, running and stair activity were 3.718%, 7.982%, and 10.348% (T1 rho) and 3.459%, 6.599%, and 8.848% (T2); the corresponding reductions rates of deep layer were 2.559%, 6.093%, and 8.631% (T1 rho) and 2.245%, 2.738%, and 3.251% (T2).

### Tibiofemoral cartilage

According to an analysis of variance for random block design data (Table 2), the T1 rho values for femoral and tibial cartilage significantly differed (P<0.05) after walking, running and stair activity, except for PMFC and PLFC, and the T2 values for femoral and tibial cartilage significantly differed (P<0.05) except for the middle part of CMFC, the anterior part of MTC, PMFC and PLFC.

**Table 2:** ANOVA for random block design data and bonferroni test of patellofemoral cartilage among rest, walking, running and stair activity.

Cartilage	T1 rho								T2 mapping							
	F value	P value	1 vs 2	1 vs 3	1 vs 4	2 vs 3	2 vs 4	3 vs 4	F value	P value	1 vs 2	1 vs 3	1 vs 4	2 vs 3	2 vs 4	3 vs 4
MFC																
Anterior part	13.42	<0.001	0.342	<0.001	<0.001	0.068	0.002	1.000	6.28	0.001	1.000	0.007	0.003	0.217	0.107	1.000
Middle part	14.18	<0.001	0.130	<0.001	<0.001	0.005	0.061	1.000	2.12	0.106	1.000	0.251	0.317	0.827	1.000	1.000
Posterior part	15.17	<0.001	0.172	<0.001	<0.001	0.004	0.013	1.000	14.07	<0.001	0.120	<0.001	<0.001	0.253	0.002	0.582
PMFC	2.37	0.079	1.000	1.000	0.063	1.000	0.614	0.978	1.05	0.379	0.635	1.000	1.000	1.000	1.000	1.000
MTC																
Anterior part	9.51	<0.001	0.183	<0.001	0.001	0.041	0.559	1.000	2.17	0.100	1.000	1.000	0.086	1.000	0.752	0.749
Middle part	17.77	<0.001	0.011	<0.001	<0.001	0.319	0.003	0.520	7.88	<0.001	0.668	0.002	<0.001	0.234	0.052	1.000
Posterior part	80.26	<0.001	<0.001	<0.001	<0.001	<0.001	<0.001	0.577	71.92	<0.001	<0.001	<0.001	<0.001	<0.001	<0.001	1.000
LFC																
Anterior part	5.49	0.002	0.354	0.181	0.001	1.000	0.223	0.429	3.91	0.012	1.000	0.058	0.031	0.495	0.302	1.000
Middle part	12.65	<0.001	0.365	0.001	<0.001	0.291	0.001	0.366	6.01	0.001	1.000	0.315	0.001	1.000	0.023	0.232
Posterior part	15.64	<0.001	0.079	<0.001	<0.001	0.001	0.339	0.253	9.81	<0.001	0.192	<0.001	0.003	0.023	0.832	0.829
PLFC	1.23	0.305	0.866	1.000	0.471	1.000	1.000	1.000	0.52	0.672	1.000	1.000	1.000	1.000	1.000	1.000
LTC																
Anterior part	11.21	<0.001	1.000	0.095	<0.001	0.490	<0.001	0.036	4.42	0.007	1.000	0.184	0.015	0.464	0.048	1.000
Middle part	23.18	<0.001	0.010	<0.001	<0.001	0.106	<0.001	0.174	4.79	0.004	0.642	0.082	0.003	1.000	0.277	1.000
Posterior part	38.16	<0.001	<0.001	<0.001	<0.001	0.003	<0.001	0.303	22.62	<0.001	0.003	<0.001	<0.001	0.008	0.008	1.000

Note: PC = patella cartilage, TC = trochlea cartilage;

1: rest, 2: walking, 3: running, 4: stair activity.

According to the Bonferroni test for comparison of multiple samples (Table 2), only the middle and posterior part of MTC and LTC exhibited significantly different T1 rho values after walking. The T1 rho value significantly differed after running, except for the anterior part of CLFC and LTC, PMFC and PLFC. The T1 rho values after stair activity significantly differed, except for PMFC and PLFC. The T2 value after walking had statistical significance(P<0.05) only for the posterior part of MTC and LTC. The T2 value significantly differed after running, except for the middle part of CMFC, the anterior part of MTC, the anterior and middle part of CLFC and LTC, PMFC and PLFC. The T2 value significantly differed after stair activity, except for the middle part of CMFC, the anterior part of MTC, the anterior part of CLFC, PMFC and PLFC.

The central weight-bearing regions of femoral cartilage showed higher reductions in relaxation times than the posterior nonweight-bearing regions after walking, running and stair activity. The reductions of T1 rho and T2 relaxation times for posterior nonweight-bearing regions after physiological activities were not significant.

### Similar and different influences of the various exercises on knee cartilage

#### Similar influences

**Fig 3:**
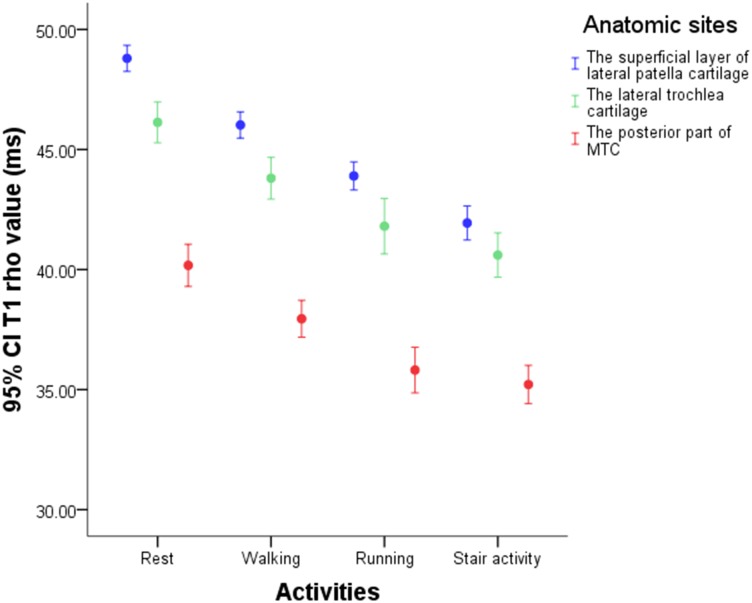
The T1 rho values of the superficial layer of lateral patella cartilage, the lateral trochlea cartilage and the posterior part of medial tibial cartilage.

**Fig 4:**
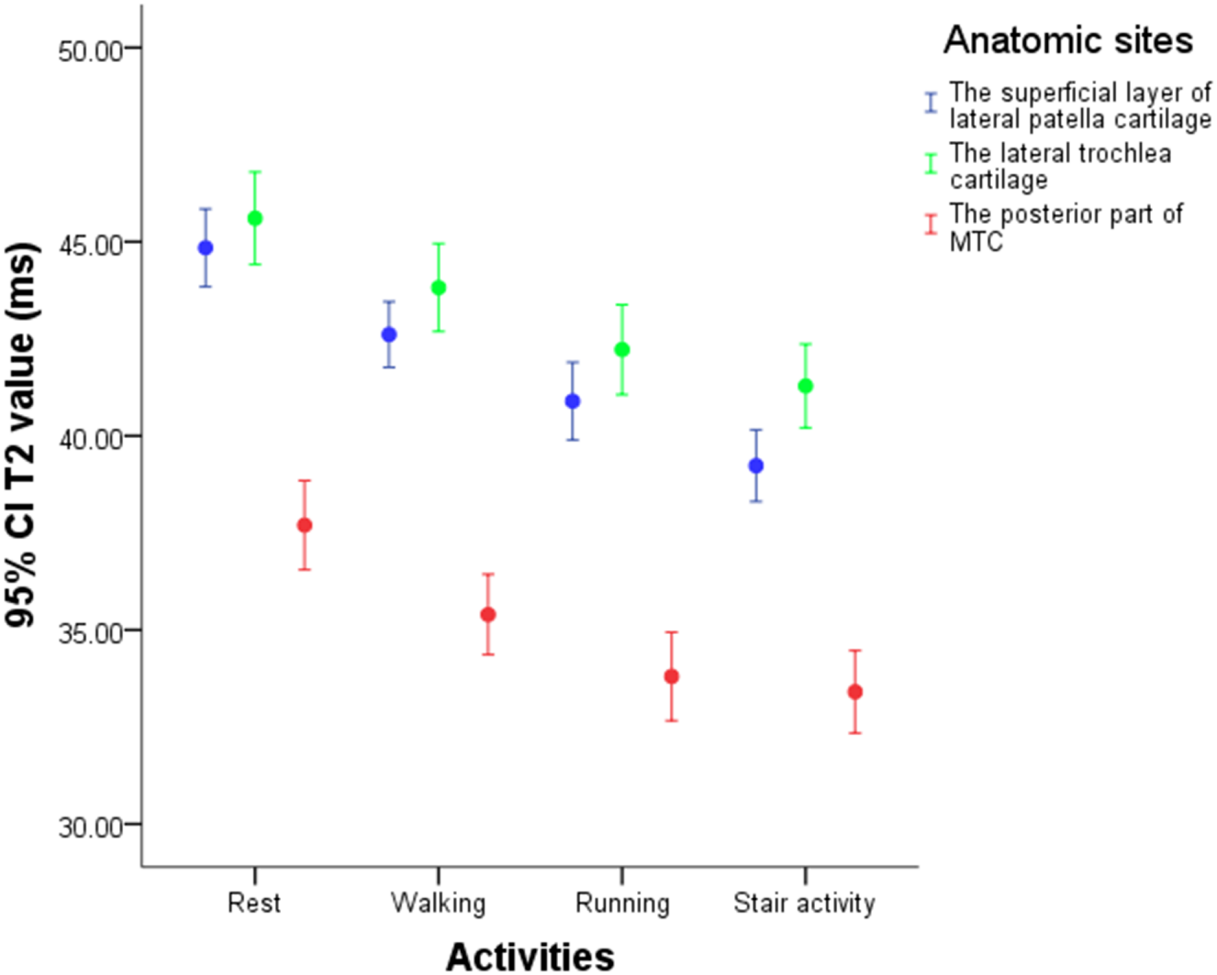
The T2 values of the superficial layer of lateral patella cartilage, the lateral trochlea cartilage and the posterior part of medial tibial cartilage.

T1 rho and T2 values after walking, running and stair activity had reduction for all regions of knee cartilage, especially in the superficial layer of lateral patella cartilage, the lateral trochlea cartilage and the posterior part of medial tibial cartilage (Figs 3 and 4). After walking, T1 rho of the three regions decreased by 5.667%, 5.031%, and 5.491%; T2 decreased correspondingly by 4.923%, 3.889%, and 6.060%. After running, the corresponding values were 10.010%, 9.396%, and 10.752% (T1 rho) and 8.741%, 7.396%, and 10.267% (T2). The reduction rates after stair activity were 14.052%, 11.960%, and 12.223% (T1 rho) and 12.407%, 9.407%, and 11.297% (T2).

Compared with rest values, the reduction rates in cartilage after exercise were ranked in the following order: patellofemoral cartilage> medial tibiofemoral cartilage> lateral tibiofemoral cartilage. The reduction rates for three compartments after walking were 3.455%, 3.024%, and 2.901% (T1 rho) and 3.094%, 2.362%, and 1.657% (T2); after running, the values were 7.541%, 6.657%, and 4.847% (T1 rho) and 5.376%, 4.109%, and 3.174% (T2); and after stair activity, the values were 9.842%, 7.298%, and 6.309% (T1 rho) and 6.914%, 5.038%, and 3.972% (T2).

Compared with rest values, T1 rho and T2 reductions in patellofemoral cartilage were ranked as follows: lateral part> middle part> medial part. The reduction rates for the lateral, middle and medial parts of patellofemoral cartilage after walking were 4.931%, 3.263%, and 2.048% (T1 rho) and 3.916%, 2.987%, and 2.282% (T2); the corresponding reductions after running were 9.029%, 7.661%, and 5.777% (T1 rho) and 6.599%, 4.865%, and 4.505% (T2); and the corresponding reductions after stair activity were 12.111%, 9.491%, and 7.706% (T1 rho) and 9.013%, 6.018%, and 5.444% (T2).

Compared with rest values, the reductions in T1 rho and T2 in tibiofemoral cartilage were ranked as follows: posterior part> middle part> anterior part. The reduction rates for the posterior, middle and anterior parts of tibiofemoral cartilage after walking were 3.780%, 2.810%, and 2.205% (T1 rho) and 3.264%, 1.466%, and 1.239% (T2); the corresponding reductions after running were 8.465%, 5.792%, and 4.556% (T1 rho) and 6.042%, 3.201%, and 2.955% (T2); and the corresponding reductions after stair activity were 8.252%, 6.964%, and 6.053% (T1 rho) and 6.437%, 4.282%, and 4.096% (T2).

#### Different influences

The T1 rho and T2 values of knee cartilage after stair activity decreased more than running, except for the middle and posterior parts of medial femoral cartilage and the posterior part of lateral femoral cartilage. According to the Bonferroni test for comparison between running and stair activity (Tables 1 and 2), only the superficial layer of lateral patella cartilage and the lateral trochlea cartilage exhibited significant differences (P values of <0.001 and 0.001, respectively, for T1 rho and 0.001 and 0.009 for T2, respectively).

## Discussion

During physiological activities, knee cartilage suffers pressure loading and changes in biochemical components. Fluid shifts within the cartilagematrix, and collagen fibers experience various degrees of deformation that are accompanied by relative increases in PG concentration, thus decreasing T1 rho relaxation times. Research [3,5,6,14–16] has shown that T1 rho relaxation time is negatively correlated with PG content, and T2 value is positively correlated with collagen fiber and water content. Collagen fiber deformation and water loss lead to decreases in T2 relaxation times [5,6,8]. Load and stress incurred during walking are much lower than those incurred during running and stair activities.

The superficial layers of lateral patella cartilage, the lateral trochlea cartilage and the posterior part of medial tibial cartilage experienced the greatest reductions among the knee cartilage tissues, indicating that these regions may experience greater loading and friction and were more vulnerable to damage during exercise. In addition, the material properties of these tissue are different and these regions that deform the most are more compliant. Compression loading in knee cartilage is region specific, which accounts for the nonuniform cartilage degeneration observed in the clinic.

The relaxation times measured for the superficial layer of patella cartilage showed greater decreases than the deep layer, indicating that the superficial layer is the main area that resists pressure and experiences matrix hydration and fiber deformation. Sports involving repetitive loading lead to cyclic hydraulic pressure changes in cartilage; in particular, fluid shifts and compression in the subcutaneous layer account for most pressure in the cartilage. The superficial zone of cartilage is less stiff than the deep zone [17], so the superficial is more compliant. Subburaj et a [15] determined that exercise transformed the tangentially oriented fibers in the superficial layer more than the radial fibers in the deep layer.

Patellofemoral cartilage experienced the largest loading during exercise, followed by medial tibiofemoral cartilage and lateral tibiofemoral cartilage. Patellofemoral cartilage is subjected to greater compressive stress during stair activity than during running, particularly in the superficial layer of lateral patella cartilage and the lateral trochlea cartilage. Other studies report values ranging from 229 to 4000 N for patellar contact force, 1.3 to 13.8 MPa for patellar contact (compressive) stress and 0.6 to 2.5 MPa for octahedral shear stress [18–21]; these values increase friction between patellar cartilage and articular surfaces. Recent research [22] indicates that frequent knee bending is associated with a higher prevalence of knee cartilage lesions (particularly in patellofemoral cartilage) and with an increased risk of progression of cartilage and meniscal lesions in asymptomatic middle-aged subjects. Silverberg [23] have showed regional changes in the knee joint between patellofemoral cartilage and tibiofemoral cartilage and their ties to collagen and proteoglycan structure in the tissue [24]. Tibial cartilage is more compliant and dissipates more energy closer to the articular surface than femoral cartilage. Material properties heterogeneity of cartilage is another important factor for the biomechanical difference.

The medial part of the tibiofemoral cartilage experienced a greater reduction than lateral part, demonstrating that medial cartilage experiences greater pressure and is more likely to suffer structural damage. Zarins et al [3] stated that in the normal state, 60–80% of the total intrinsic compressive load was transmitted to the medial cartilage in the knee. Temple et al [25] noted that age-associated cartilage deterioration occurred at an earlier age in the MFC than in the LFC. The medial and lateral femoral condyles are shaped differently. The antero-posterior axial part of the lateral femoral condyle is in sagittal alignment, but those of the medial femoral condyle lies at an angle of approximately 22° to the sagittal alignment. When the knee bends, the lateral femoral condyle is higher than the medial femoral condyle. When the tibiofemoral joint shares longitudinal compressive stress, the femoral condyles impact the tibial plateau, and the tibiofemoral joint experiences introversive stress, which increases the compressive loads on the medial tibial plateau.

The load distribution of patellofemoral cartilage exhibits the following trend: lateral part> middle part> medial part. When the knee bends 20 degrees, patella cartilage slips into the trochlear cartilage groove, with the lateral part contacting first. In the range from 20 degrees to 90 degrees, the lower pole of patella cartilage contacts trochlea cartilage, and the medial part is not in contact. Until the blending angle is greater than 90 degrees, medial patella cartilage contacts medial trochlea cartilage. Lateral patella cartilage is the main area that bears load during knee reciprocating flex movement as the stress center of leverage. Akbarshahi [26] calculated that the contact force acting on the lateral facet of the patella was 4–6 times higher than that acting on the medial facet. So the lateral cartilage of the patellofemoral joint is more prone to suffer injury and degeneration than medial cartilage.

The stress characteristics of tibiofemoral cartilage also follow a certain trend: posterior part> middle part> anterior part. According to some researchers [27–30], the tibial plateau is not perpendicular to the longitudinal axis of the tibial diaphysis, and a tibial posterior slope angle (PSA) is present. Due to the PSA, the femoral condyle has a tendency to slide backwards under axial loads. Zhang WG et al [31] stated the areas of stress on the tibiofemoral joint under 30–45° flexion lie in the middle of the tibial plateau and that the areas of stress move posteriorly when the knee is bent at angles of greater than 60°. Other researchers [30,31] have reported different PSA values between the medial tibial plateau and the lateral tibial plateau, but they have consistently stated that the medial PSA is greater than the lateral PSA; this explains why the posterior part of the medial tibial cartilage takes most of the load in the tibiofemoral joint.

The different changes observed for the T1 rho and T2 relaxation times of patellofemoral cartilage and tibiofemoral cartilage after running and stair activity suggested that the middle and posterior parts of medial femoral cartilage and the posterior part of lateral femoral cartilage experienced greater pressure after running than after stair activity, whereas the patella, trochlea and tibial cartilage experienced more load after stair activity than after running. Stair activity affects patellofemoral cartilage to a greater extent than tibiofemoral cartilage.

The results also suggest that T1 rho relaxation time may be more sensitive than T2 values for detecting knee articular cartilage changes after walking, running and stair activity. Wang reported that T1 rho relaxation times appeared more sensitive than T2-weighted imaging when differentiating between normal cartilage and early-stage osteoarthritis [32,33]. Greater changes in T1 rho values upon loading may be related to the fact that knee cartilage has abundant PGs, to which T1 rho relaxation time is sensitive, while T2 relaxation time is poorly correlated with PG content.

Our study has several limitations. First, too few subjects were included, and a large sample size is needed to increase confidence. Second, T1 rho sequences and T2 mapping were not determined at the same time, which inevitably causes error. Third, the meniscus and alignment were not analyzed during the research, and these are important factors in the biomechanical mechanism underlying knees. Finally, the research was conducted in vivo and lacked the use of histopathology as the gold standard.

The study demonstrated that fluid shifts, collagen fiber deformation, spatial heterogeneity, inherent differences in material properties and tissue stiffness had close relationship with cartilage loading characteristics. The patellofemoral cartilage was the main load bearing area and stress fulcrum of the reciprocating flexion movement in physiological activities, and stair activity affected patellofemoral cartilage to a greater extent than tibiofemoral cartilage, while femoral cartilage was more influenced by running than stair activity.

## Supporting information

S1 Table Participants information. (XLS)

S2 Table Table T1 rho of patellofemoral cartilage in rest, walking, running and stair activity. (XLS)

S3 Table T2 mapping of patellofemoral cartilage in rest, walking, running and stair activity. (XLS)

S4 Table T1 rho of tibiofemoral cartilage in rest, walking, running and stair activity. (XLS)

S5 Table T2 mapping of tibiofemoral cartilage in rest, walking, running and stair activity. (XLS)
